# Factors associated with the growing-finishing performances of swine herds: an exploratory study on serological and herd level indicators

**DOI:** 10.1186/s40813-018-0082-9

**Published:** 2018-03-22

**Authors:** C. Fablet, N. Rose, B. Grasland, N. Robert, E. Lewandowski, M. Gosselin

**Affiliations:** 1 Agence Nationale de Sécurité Sanitaire de l’alimentation, de l’environnement et du travail (Anses), Laboratoire de Ploufragan/Plouzané, Unité Epidémiologie et Bien-Etre du Porc, B.P. 53, 22440 Ploufragan, France; 2 Agence Nationale de Sécurité Sanitaire de l’alimentation, de l’environnement et du travail (Anses), Laboratoire de Ploufragan/Plouzané, Unité Génétique Virale et Biosécurité, B.P. 53, 22440 Ploufragan, France; 3 Boehringer Ingelheim France - Santé Animale, Les Jardins de la Teillais, 3 allée de la grande Egalonne, 35740 Pacé, France; 4 Univet Santé Elevage, rue Monge, 22600 Loudéac, France; 5 Université Bretagne-Loire, Cité internationale 1 place Paul Ricoeur CS 54417, 35044 Rennes, France

**Keywords:** Herd technical performance, Management, PRRS, PCV2

## Abstract

**Background:**

Growing and finishing performances of pigs strongly influence farm efficiency and profitability. The performances of the pigs rely on the herd health status and also on several non-infectious factors. Many recommendations for the improvement of the technical performances of a herd are based on the results of studies assessing the effect of one or a limited number of infections or environmental factors. Few studies investigated jointly the influence of both type of factors on swine herd performances. This work aimed at identifying infectious and non-infectious factors associated with the growing and finishing performances of 41 French swine herds.

**Results:**

Two groups of herds were identified using a clustering analysis: a cluster of 24 herds with the highest technical performance values (mean average daily gain = 781.1 g/day +/− 26.3; mean feed conversion ratio = 2.5 kg/kg +/− 0.1; mean mortality rate = 4.1% +/− 0.9; and mean carcass slaughter weight = 121.2 kg +/− 5.2) and a cluster of 17 herds with the lowest performance values (mean average daily gain =715.8 g/day +/− 26.5; mean feed conversion ratio = 2.6 kg/kg +/− 0.1; mean mortality rate = 6.8% +/− 2.0; and mean carcass slaughter weight = 117.7 kg +/− 3.6). Multiple correspondence analysis was used to identify factors associated with the level of technical performance. Infection with the porcine reproductive and respiratory syndrome virus and the porcine circovirus type 2 were infectious factors associated with the cluster having the lowest performance values. This cluster also featured farrow-to-finish type herds, a short interval between successive batches of pigs (≤3 weeks) and mixing of pigs from different batches in the growing or/and finishing steps. Inconsistency between nursery and fattening building management was another factor associated with the low-performance cluster. The odds of a herd showing low growing-finishing performance was significantly increased when infected by PRRS virus in the growing-finishing steps (OR = 8.8, 95% confidence interval [95% CI]: 1.8–41.7) and belonging to a farrow-to-finish type herd (OR = 5.1, 95% CI = 1.1–23.8).

**Conclusions:**

Herd management and viral infections significantly influenced the performance levels of the swine herds included in this study.

**Electronic supplementary material:**

The online version of this article (10.1186/s40813-018-0082-9) contains supplementary material, which is available to authorized users.

## Background

Swine farm profitability and efficiency rely in part on technical performance, which in turn depends on pig health and welfare. Several infectious respiratory or digestive pathogens can reduce swine performance during the growing-finishing steps. The porcine reproductive and respiratory syndrome virus (PRRSV), porcine circovirus type 2 (PCV2), swine influenza A viruses (swIAV), *Mycoplasma hyopneumoniae* (*M. hyopneumoniae*) and *Lawsonia intracellularis (L. intracellularis*) are among the main infectious pathogens causing, alone or in combination, marked economic losses to the swine industry throughout the world [1–4]. *Mycoplasma hyopneumoniae* is the aetiological cause of enzootic pneumonia and is considered to be one of the primary pathogens involved in the porcine respiratory disease complex (PRDC) together with PRRSV, PCV2 and/or swIAV [3, 5]. PCV2 also contributes to other syndromes collectively known as porcine circovirus-associated diseases [6], whereas PRRSV alone is responsible for reproductive failures in pregnant sows, high pre-weaning mortality in piglets infected in utero and respiratory signs in growers and finishers [7–9]. Proliferative enteropathy caused by *L. intracellularis* is another current disease – but targets the digestive tract – having considerable impact on pig production and herd economics [10].

Non-infectious factors also directly drive herd performance through diet and climatic conditions, or indirectly by affecting the occurrence and severity of diseases [11, 12]. Non-infectious environmental factors act on the pathogen load (i.e. the amount of micro-organisms to which the pig is exposed), the intensity and frequency of pathogen exposition, and on the pig, by modulating the defence mechanisms through which the pig handles the pathogen challenge [13]. Disease outcome in turn depends on the balance between the pathogen pressure and the pig’s ability to cope with them. In modern swine production systems, multiple environmental factors may interfere with this delicate balance and need to be considered and adapted to reduce disease incidence and severity and thus enhance farm profitability.

Many recommendations for the improvement of the technical performances of a herd are based on the results of studies assessing the effect of one or a limited number of pathogens or environmental factors. To date, few studies have simultaneously investigated both types of factor on swine herd performance. This situation may be related to the difficulty of running studies obtaining valid findings. Effective and valuable recommendations indeed rely on valid results that allow inference about the associations to the target population. Obtaining reliable data is a crucial and challenging issue that needs to be properly considered in observational studies. Data collection should be designed in order to reduce potential bias and ensure the validity of the measures. Questionnaires are one of the most commonly used tools for collecting data, particularly related to environmental factors, in veterinary epidemiology [14]. The information validity of data obtained by questionnaire should be assessed whenever possible [15]. Hence, in questionnaire-based survey, it is advised to combine interviews with people working on farm with direct observation during an on-farm visit so as to decrease misclassification bias. Compliance with the reported measures is another tricky point that may lead to information bias and which is the hardest to assess [16]. Dealing with diagnostic tests used to describe infectious factors, imperfect diagnostic procedures could also represent a source of bias. The assays should have previously been assessed and validated under experimental and field conditions in order to adjust the results according to the diagnostic performances and to control misclassification bias. The aim of our study was to identify infectious and non-infectious factors associated with the technical performance of the growing-finishing steps in a sample of 41 herds.

## Methods

### Study design

Data and sera used were collected from 41 French pig farms involved in a study on the course of PCV2 infection (western France 2014–2015). The study was carried out in subclinically PCV2-infected herds without piglet vaccination against this virus. The herds were provided by the veterinarians at ‘Univet santé élevage’ and ‘Cybelvet’ veterinary clinics. Blood was sampled from 20 pigs selected at random from two batches in each herd (10 pigs 10–12 weeks old and 10 pigs at least 22 weeks old). Data on management, biosecurity and farm practices were collected via a questionnaire that was filled out with the farmer. The questionnaire is available upon request (in French, 26 closed or semi-closed questions). The main technical performance values of the growing-finishing steps (average daily weight gain from 8 to 115 kg [ADG], feed conversion ratio from 8 to 115 kg [FCR], mortality from 8 to 115 kg [MORT] and carcass slaughter weight [CSW] in 2014) were obtained from the technical-economic database managed by the French Pork and Pig Institute (IFIP).

### Laboratory analyses

Serum samples from all pigs were tested for antibodies against *L. intracellularis* (SVANOVIR L.intracellularis/Ileitis-Ab, Boehringer Ingelheim Svanova, Sweden, successor of the bioScreen Ileitis Antibody ELISA with sensitivity (Se) ranging from 72 to 96.5% and specificity (Sp) from 83 to 100%; [17–19]), *M. hyopneumoniae* (*Mycoplasma hyopneumoniae* ELISA, OXOID Ltd., UK; formely DAKO ELISA; with Sp = 100% and Se ranging from 49% to 100% according to experimental trials [20–23]) and PCV2 (SERELISA® PCV2 Ab Mono Blocking, Synbiotics Europe, France, Se = 86% and Sp = 85% [24]). A serum sample was considered positive for *L. intracellularis* antibodies if the percentage inhibition was ≥30% [18]. Any serum sample presenting a percentage inhibition was > 50% was considered positive for *M. hyopneumoniae* antibodies [22, 23]. A serum sample was classified as positive for PCV2 antibodies if the SERELISA® titer was > 170 ELISA units [24].

Pools of 5 samples were constituted and analysed to detect PRRSV antibodies (IDEXX PRRS X3 Ab Test, IDEXX, USA, Se = 97.5% and Sp = 100% of pool of 5 serum samples from growing-finishing pigs [25]). A pool was considered positive when the sample to positive control (S/P) optical density ratio was ≥0.4. Antibodies against swIAV were detected in the serum samples of the oldest pigs (*n* = 6 samples/herd) using a commercial ELISA kit (ID Screen® Influenza A antibody competition, IDVet, France, Se = 69% and Sp = 89%, [26]). A serum sample was classified positive for swIAV antibodies if the percentage inhibition was ≥60% [27].

### Statistical analysis

#### Definition of the outcome (the level of herd growing-finishing performances) and explanatory variables

Associations between the four parameters describing the technical performance (ADG, FCR, MORT, CSW) of the growing-finishing pigs in the sampled herds were investigated using principal component analysis. The main objective in principal component analysis is to detect associations within a set of continuous variables in a small number of dimensions and to provide a low-dimensional (often two-dimensional) graphical representation of these associations [28]. Each variable is represented by an arrow (eigenvector) inside a correlation circle, the longer the length of the arrow, the higher the contribution of the variable to the inertia. The angle between arrows indicates the degree of correlation between the variables; the smaller the angle, the higher the correlation. An angle of 90° indicates that the two variables are independent and an angle of 180° shows a negative correlation.

The parameters describing technical performance were then included in a clustering analysis to identify the groups differing in performance. This classification process leads to clusters of herds based on the degree of similarity between the samples with regard to the variables. Two groups of herds were formed. A *t*-test was used to compare ADG and CSW between the two groups (*p* < 0.05) and a Kruskal-Wallis test was used to compare FCR and MORT between the groups (p < 0.05). These two groups were thereafter used as a dichotomous outcome variable to assess the relationships between infectious and non-infectious factors associated with the level of growing-finishing performance of the herds.

All the explanatory variables related to the infectious agents were classified into two or more categories according to the frequency of pigs positive for a given pathogen per age category and/or at the herd level when applicable. The cut-off points were determined according to the distribution of variables. The serological status of the batch and/or the herd to a given pathogen was also considered when relevant. A unit (herd or batch) was classified as positive when at least one sample tested positive. Regarding PCV2, the laboratory analyses leading to semi-quantitative results, i.e. titer expressed as ELISA units [29], the frequency of pigs with high antibody titers was also calculated and categorized. For all variables, the number of categories was limited to ensure minimal category frequencies of 10%. Since PRRS natural infection were known to occur in PRRS vaccinated herds (data from veterinarians in charge of the herd health management, personal communications), vaccinated herds were considered as PRRS-seropositive herds. Description of these explanatory variables is given Table 1. All the variables related to non-infectious factors were categorical variables.

**Table 1:** Description of the categorical explanatory variables related to the serological status to bacterial and viral pathogens

Definition of the variables	% herds per level
% of pigs seropositive to *Lawsonia intracellularis* after 16 weeks of age	
< 60%	26.83
≥ 60%	73.17
Pigs seropositive to *Lawsonia intracellularis*	
before 16 weeks of age	51.22
after 16 weeks of age	48.78
% of finishing pigs seropositive to *Lawsonia intracellularis*	
< 50%	60.98
≥ 50%	39.02
Serological status to *Mycoplasma hyopneumoniae*	
Negative	55
Positive (at least one positive sample)	45
Serological profile to *Mycoplasma hyopneumoniae*	
Negative	55
Positive before 16 weeks of age (at least one positive sample)	37.5
Positive after 16 weeks of age (at least one positive sample)	7.5
% of pigs with antibodies against *Mycoplasma hyopneumoniae* before 16 weeks of age	
≤ 20%	80.49
> 20%	19.51
% of pigs with antibodies against *Mycoplasma hyopneumoniae* before 16 weeks of age	
≤ 10%	73.17
> 10%	26.83
% of pigs with antibodies against *Mycoplasma hyopneumoniae* after 16 weeks of age	
≤ 10%	80.49
> 10%	19.51
% of pigs with antibodies against *Mycoplasma hyopneumoniae* during the finishing phase	
≤ 10%	78.05
> 10%	21.95
% of pigs with antibodies against swine Influenza A virus	
≤ 20%	56.1
> 20%	43.9
% of pigs with antibodies against swine Influenza A virus	
≤ 80%	70.73
> 80%	29.27
Serological status to swine Influenza A virus	
Negative	46.34
Positive (at least one positive sample)	53.66
Antibodies against PRRSV before 16 weeks of age	
No	80.49
Yes (at least one positive pool)	19.51
Antibodies against PRRSV after 16 weeks of age	
No	56.1
Yes (at least one positive pool)	43.9
Serological status to PRRSV	
Negative	56.1
Positive (at least one positive pool)	43.9
Serological profile to PRRSV	
Negative	56.1
Positive before 16 weeks of age (at least one positive pool)	19.51
Positive after 16 weeks of age (at least one positive pool)	24.39
% of pigs with antibodies against Porcine Circovirus Type 2 (PCV2) before 16 weeks of age	
≤ 50%	29.27
> 50%	70.73
% of pigs with antibodies against Porcine PCV2 after 16 weeks of age	
≤ 70%	19.51
> 70%	80.49
Anti-PCV2 IgG antibody titers > 5000 ELISA units before 16 weeks of age	
No	85.37
Yes (at least one pig)	14.63
Anti-PCV2 IgG antibody titers > 5000 ELISA units after 16 weeks of age	
No	31.71
Yes (at least one pig)	68.29
Anti-PCV2 IgG antibody titers > 5000 ELISA units during the fattening phase	
No	31.71
Yes (at least one pig)	68.29
> 10% of pigs with a SERELISA® titer > 5000 ELISA Units for antibodies against PCV2	
No	41.46
Yes	58.54
> 20% of pigs with a SERELISA® titer > 5000 ELISA Units for antibodies against PCV2	
No	51.22
Yes	48.78

#### Associations between the level of growing-finishing performances and the infectious and non-infectious factors

A two-step procedure was used to assess the relationships between the explanatory variables and the level of herd growing-finishing performance. The first step was based on a univariable analysis relating the outcome variable to each explanatory variable. Only factors associated with the level of growing-finishing performance (likelihood ratio *χ*^2^-test, *p* < 0.15) were selected for a multivariable analysis.

The second step involved a multiple correspondence analysis that included all factors that had passed the first screening step. The main objective in multiple correspondence analysis is to detect the associations within a set of categorical variables in a small number of dimensions and to provide a low-dimensional (often two-dimensional) graphical representation of these associations [30, 31]. The FactoMineR package for R was used [32]. The effects of the explanatory factors on the level of technical performances were then quantified by performing a logistic-regression analysis. All selected explanatory variables were checked for multicolinearity (*χ*^2^-test, *p* < 0.05), and those most strongly associated with the outcome variable and having biological relevance were selected. The logistic regression was performed according to the method described in Hosmer and Lemeshow [33] (PROC LOGISTIC, SAS 9.1, SAS Inst., Cary, NC, USA). A backward stepwise procedure was used to select the variables that were significantly (*p* < 0.05) associated with the outcome variable. At each step, the variable with the highest *p*-value was removed from the model. This procedure was continued until all variables were significant (*p* < 0.05). The odds ratio and 95% confidence intervals were calculated from the final logistic model. Goodness-of-fit for the final model was assessed using the Pearson *χ*^2^, deviance and Hosmer-Lemeshow goodness-of-fit [33].

## Results

### Features of the study sample

The herds were located in western France. In all, 20 herds were farrow-to-finish with on average 217 sows (standard deviation [SD]: 149 sows) and 21 herds were weaning-to-finishing farms (on average 3769 pigs, SD = 1413 pigs). Replacement stock and sows were vaccinated against PCV2 in 31.7% of the herds and 24.4% of the herds only vaccinated gilts against this virus. Growing pigs were not vaccinated against swIAV or PCV2 in any of the herds. In 73.2% and 19.5% of the herds, piglets were vaccinated against *M. hyopneumoniae* or PRRS respectively.

### Relationships between the technical parameters

**Fig. 1:**
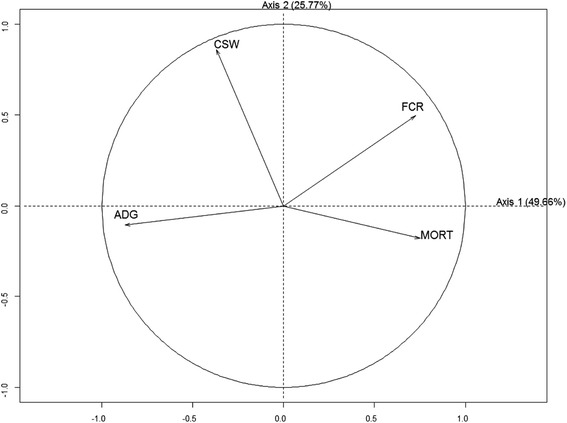
Principal component analysis describing associations between average daily weight gain from 8 to 115 kg (ADG), feed conversion ratio from 8 to 115 kg (FCR), mortality from 8 to 115 kg (MORT) and carcass slaughter weight (CSW) (41 French pig farms, western France, 2014–2015)

The principal component analysis revealed one group of positively correlated variables (right side of the map) describing the feed conversion ratio from 8 to 115 kg and the mortality from 8 to 115 kg (Fig. 1). These variables were negatively correlated with average daily gain from 8 to 115 kg. The carcass slaughter weight was not correlated with the other three variables.

### Clusters of herds related to growing-finishing performance

Two groups of herds were identified by the clustering analysis: a cluster of 24 herds with the highest technical performance values (group 1) and a cluster of 17 herds with the lowest performance values (group 2) (Table 2).

**Table 2:** Technical characteristics of the whole sample and the two identified groups with different levels of growing-finishing performance as defined by the hierarchical cluster analysis (mean and standard deviation [sd])

	Overall sample (41 herds)		Group 1 (24 herds)		Group 2 (17 herds)		*p*-value^a^
	mean	sd	mean	sd	Mean	sd	
Average daily weight gain from 8 to 115 kg (g/day)	754.00	41.61	781.08	26.28	715.76	26.50	< 0.01
Feed conversion ratio from 8 to 115 kg (kg/kg)	2.53	0.13	2.48	0.08	2.60	0.14	< 0.01
Mortality from 8 to 115 kg (%)	5.21	1.99	4.09	0.93	6.79	2.03	< 0.01
Carcass slaughter weight (kg)	119.78	4.91	121.22	5.21	117.75	3.58	< 0.01

^a^Comparison between group 1 and group 2, Kruskal-Wallis test, *p* < 0.05

### Factors associated with the levels of growing-finishing performance

**Fig. 2:**
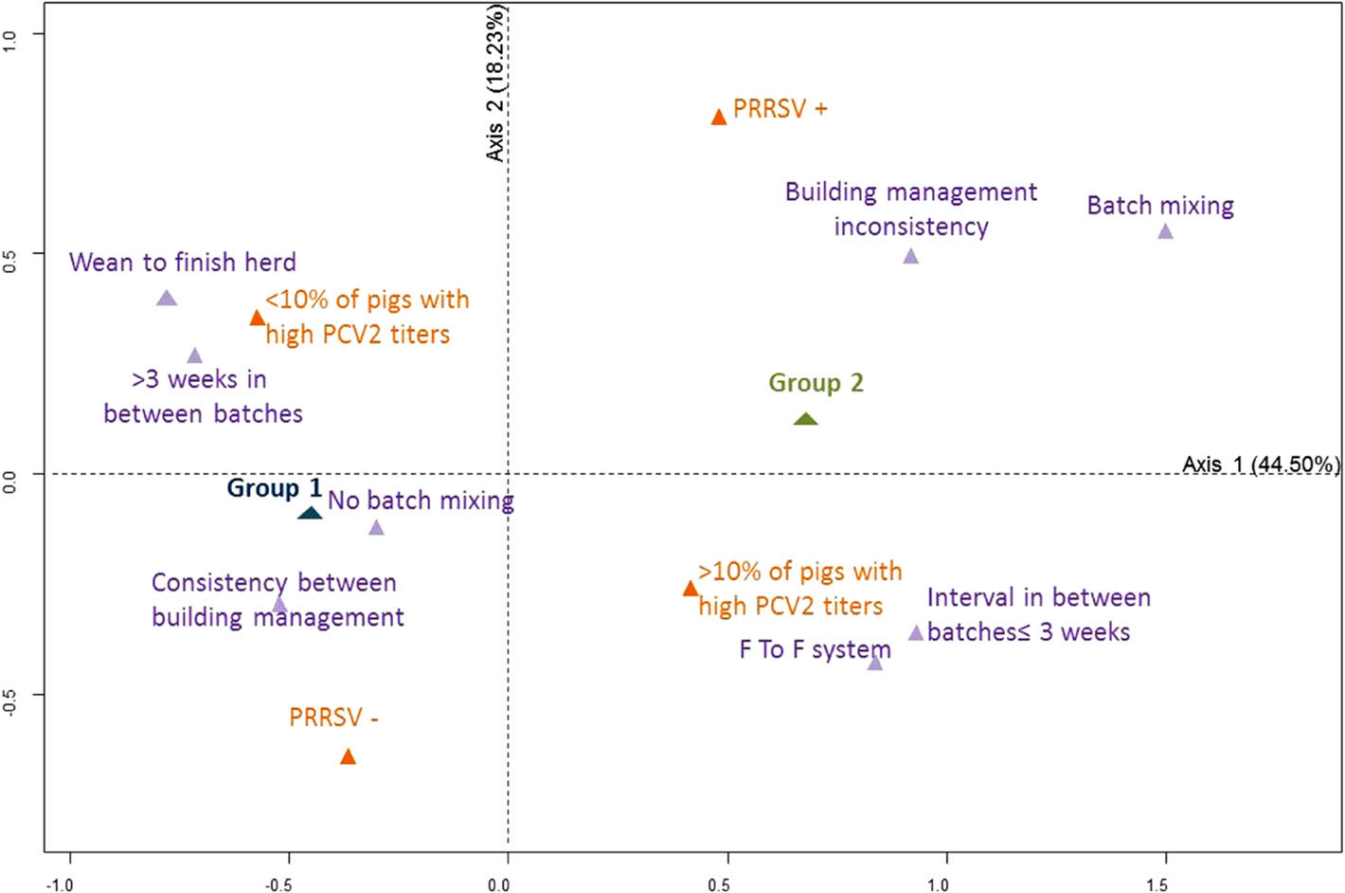
Multiple correspondence analysis describing associations between the level of growing-finishing performance and infectious and non-infectious factors (41 herds, western France, 2013–2014). Group 1: Herds having the highest growing-finishing performance values; Group 2: herds having the lowest growing-finishing performance values; PRRSV -: PRRSV seronegative infection status of growers and finishers; PRRSV +: PRRSV seropositive infection status of growers and finishers; %PCV2 titers low: < 10% of pigs with a SERELISA® titer> 5000 ELISA Units for antibodies against PCV2; %PCV2 titers high: > 10% of pigs with a SERELISA® titer > 5000 ELISA Units for antibodies against PCV2; F To F system: Farrow-to-finish herds

The variables included in the study are presented in Additional file 1. In the univariable analysis, 14 variables were associated (*p* < 0.15) with the level of herd growing-finishing performance (Additional file 1). Of these variables, 6 were included in the final multiple correspondence analysis (Fig. 2). PRRSV infection and a frequency of pigs with high antibody titers (> 5000 ELISA units) > 10% were associated with the cluster having the lowest performance values (group 2). This cluster was also characterised by farrow-to-finish-type herds and a short interval between successive batches of pigs (≤3 weeks). Mixing the pigs in the growing or/and finishing steps and inconsistency between the nursery and the fattening building management (the size of the fattening rooms do not fit well with the size of the batch of piglets coming from the nursery to follow a strict all-in-all-out management at the fattening room level, i.e. without mingling pigs from different batches in the same area) were other features of this low-performance cluster. In the logistic regression analysis, two factors significantly increased the odds of a herd showing low performance (group 2) (Table 3): PRRSV infection in the growing-finishing steps and being a farrow-to-finish-type herd. The Pearson *χ*^2^ (*p* = 0.72), deviance (*p* = 0.72) and Hosmer-Lemeshow (*p* = 0.94) goodness-of-fit tests indicated a good fit between the model and the observations.

**Table 3:** Final logistic regression model for factors associated with low growing-finishing performance (41 herds, odds ratio (OR) with 95% confidence interval (CI))

Variables	% of herds identified as low performers (Group 2)	OR	95% CI	*p*
Herd type				0.04
Farrow-to-finish	65.0	5.1	1.1–23.8	
Wean-to-finish	19.1	–		
PRRSV serological status of growers and finishers				0.01
Negative	26.1	–		
Positive	61.1	8.8	1.8–41.7	

## Discussion

In the present study, the herds were classified according to four technical parameters: average daily weight gain, feed conversion ratio, mortality rate and carcass weight of slaughtered pigs. Feed-conversion efficiency, daily weight gain and mortality are recognized as the most important production-performance factors on fattening farms [34]. All these parameters were therefore used to describe the herds according to their technical performance. Carcass slaughter weight was also taken into account because of the potential economic impact of this parameter on farmer income: farmers being partly paid according to the carcass weight. Growth performances were negatively correlated with the feed conversion ratio and mortality rate in our study. Pig growth and feed conversion efficiency have previously been shown to be correlated [34].

The study was carried out in a non-negligible, but limited, number of herds without piglet vaccination against PCV2 and without clinical signs related to PCV2-associated diseases. The results of the study may therefore only apply to this kind of herd; furthermore, our herds cannot be considered representative of this population because they were not selected at random. However, the present survey serves as an exploratory study to help design future large-scale studies, providing insight into factors associated with reduced herd performance in the growing-finishing steps in herds without obvious clinical signs of PCVD and without PCV2 vaccination of piglets. These factors can be further investigated in prospective observational studies to assess the time sequence of events. In cross-sectional studies, the outcome and potential risk factors are measured at one particular time point, making the temporality and the separation of cause and effects difficult to ascertain. In the present study, data on the technical performance as well as non-infectious factors were collected in the year prior to data collection on the infectious variables, all assayed at one time point. This study design is well-suited for examining invariable characteristics (factors that are consistent in time and not influenced by the presence or absence of the disease) [35, 36], and, accordingly, our data were mainly related to herd management, hygiene and biosecurity measures, which are believed to be relatively constant over time [37]. All data were collected by the veterinarians in charge of the herd health management plan, against which the farmer’s answers to the questionnaire could be compared. The infectious status of the herds regarding several respiratory or digestive pathogens was established by laboratory analysis performed on the samples collected during the study. The results were compared with the veterinarians’ knowledge of the herd health status and discrepancies were checked and corrected when needed if recent infections had occurred. Misclassification bias of infectious status was thus reduced.

Serology is an efficient tool widely used to describe exposure to pathogens in swine field studies [3, 38–41]. Most of the assays being generally imperfects, association of different tests such as serological and molecular techniques is recommended to increase diagnostic accuracy [3]. We solely used ELISAs in our exploratory study to determine the infectious status of the batches and herds. The ability to accurately identify associations between infectious pathogens and herd performance in growing finishing steps may thus have been reduced. On the other hand, imperfect diagnostic procedures could also represent a source of information bias. Most of the ELISAs used showed reasonable to high diagnostic performances which may have limited incorrect classification of infectious statuses. However, the results should be considered as preliminary risk indicators that pave the way for future large scale studies combining different diagnostic tests to further assess the infectious and immune statuses of the animals in regards to the growing and finishing performance.

Vaccination may impair the interpretation of serological results when the test does not differentiate antibodies against natural infection from those induced by commercial vaccines. In our study, most of the herds vaccinated against *M. hyopneumoniae* and in a fewer extent against PRRS. For *M. hyopneumoniae*, antibodies detectable in the serum of fattening pigs were found to be indicative of a recent infection independently from vaccination history, thus validating the usefulness of *M. hyopneumoniae* ELISA in our study limited to the fattening step [42, 43]. Herds where piglets were vaccinated against PRRS were considered as positive in the analyses in order to avoid a reduction in study power and because veterinarians in charge of the herd health management plan confirm that field strains were circulating.

Here, viral infections, particularly PRRSV and PCV2 infections were associated with decreased growing-finishing performance. Both viruses may be responsible, alone or in association with other infectious pathogens, for reduced technical and economical performances of infected herds having clinical or subclinical signs associated with these infections. Decreased performance is generally due to higher mortality and/or feed conversion efficiency and/or reduced daily weight gain [44, 45]. Infections by these viruses are also involved in the porcine respiratory disease complex (PRDC), one of the most costly diseases for the swine industry worldwide. Nevertheless, in our study, only PRRSV infection significantly increased the odds of reduced performance. This infection may therefore have a stronger impact on growing-finishing performance in herds without clinical signs of PCVD in western France than the PCV2 infection.

Even though earlier studies showed that growth performance of pigs subclinically infected with *L. intracellularis* is poor [46], the level of infection by *L. intracellularis* was not associated with lower herd performance in our study. Several other pathogens may disturb the gut health, particularly at the weaning age. Further studies involving the main frequent digestive pathogens in growers and finishers are needed to better assess the impact of these infections on the herd performances.

*M. hyopneumoniae* is the primary pathogen of enzootic pneumonia, a chronic respiratory disease in pigs leading to decreased performance [3]. However *M. hyopneumoniae* was not found as a main infectious pathogen associated with the cluster of lower performance. In herds clinically affected by *M. hyopneumoniae*, the seroprevalence level is generally high in the fattening unit [39, 47]. The frequency of seropositive pigs was quite low in our study suggesting a low incidence of *M. hyopneumoniae* infection in the sampled herds and a limited impact of *M. hyopneumoniae* infection on the growing and finishing performance. However, serological results alone lack of sensitivity for the diagnosis of *M. hyopneumoniae*. They have to be combined with a second parameter as clinical signs or detection of the micro-organism from samples. The detection of *M. hyopneumoniae* by PCR techniques from a variety of samples is seen as a highly sensitive tool [3]. It should thus be used in combination with serological assays in further large scale study assessing the impact of *M. hyopneumoniae* infection on growing-finishing performance.

Non-infectious factors related to farm characteristics and management practices also influenced the level of herd performance. A farrow-to-finish-type herd, a short interval in between batches, inconsistency in building management between nursery and finishing steps and mixing pigs from different batches were all factors associated with decreased performance in the growing-finishing steps, with herd type being the only factor significantly increasing the odds of having reduced herd performance. The effects of these non-infectious factors may be linked to their impact on swine health and pathogen transmission.

The type of herd has commonly been identified as a risk factor for respiratory diseases and is also found to be associated with PRRSV seropositivity [12, 48]. However, results are not always consistent across the studies. A negative effect of the finishing system, when observed, is often associated with purchasing growing pigs from multiple sources, with little attention to disease entry and control measures. In our study, wean-to-finish herds – a feature of the high performing herd group – were associated with a single or a limited number of sources, using all-in-all-out procedures. Similarly, Cleveland-Nielsen et al. [49] showed that herd type was highly correlated with management factors, suggesting that the protective effect associated with a finishing herd may be attributable to the all-in-all-out system of production. The main explanations for the greater risk in farrow-to-finish farms generally involve the often continuous movement of animals and the close contact between sows and their offspring. Sows may be reservoirs of infectious pathogens [50, 51] and the purchase of breeding stock may lead to the introduction of pathogenic micro-organisms [52]. Furthermore, the spread of infection within the herd is favoured by the probability of contact between animals of different ages and with different immune status. Infection spread is enhanced by the continuous movement of pigs inherent in this production system, which is often coupled with poor building design and layout [53]. The multi-site production technique was in part developed to circumvent the negative impacts of one-site production rearing systems. Multi-site rearing systems are defined as any farm in which the stages of production or age groups are reared on separate sites and locations [54]. All-in-all-out management rather than continuous pig flow is required in such a system. By combining strict all-in-all-out management policies and geographical separation of the production sites, the multi-site rearing system allows to reduce or even to avoid pathogens transmission from sows to piglets and as a consequence to enhance pig performance [54]. Interestingly, rearing single-source isowean piglets in multi-site production systems was found to be beneficial for the production performance rather than rearing pigs originating from multiple sources [54].

Herd type and management practices are interrelated and their specific effects are not always easy to identify and evaluate. In our study, herd type was strongly associated with other management practices such as the interval between successive batches and mixing pigs from different batches as well as inconsistency in building management between nursery and finishing steps. Even though the logistic regression models quantified the effect of the explanatory factors on the outcome, this method cannot incorporate highly correlated factors. Multiple correspondence analysis helps overcome this limitation, even though the strength of association between each explanatory factor and the outcome is not directly quantified [55]. We thus combined both types of analysis to better describe the underlying relationships of strongly correlated explanatory variables and expand on the number of parameters that farmers and their herd health advisors can adjust. The effect of herd-type included in the final regression model may thus be considered given its relationship with other management factors and their interactions.

The effect of the interval between successive batches is a singular result of our study. Similarly, Fablet et al. [56] showed that a short interval between successive batches of pigs is a risk factor for pneumonia severity, suggesting that increasing the time interval between successive batches of pigs reduces animal movement frequency and prevents mixing pigs from successive batches with different infectious and immune statuses. Reducing the frequency of animal movements may thus lead to a more stable overall immune status of the herd than a management system with continuous animal movements. We may speculate that all management practices limiting the spread of pathogens within the herd are more likely to contribute to higher technical performance.

The effect of inconsistency in building design between the nursery and finishing steps on herd performances was identified for the first time. Inconsistency in building design between the successive growing steps is generally associated with mixing pigs from different pens within a batch and/or mixing pigs between batches in the same room or even pens. In our study, mixing pigs between batches was positively correlated with inconsistency between building management. Several studies indicate that the lack of all-in-all-out management and mixing pigs during the production stages negatively affect the respiratory health or favoured respiratory infections [49, 57, 58]. On the other hand, movements are usually associated with the practice of regrouping pigs and hierarchical fights generally occur after mingling. All these conditions are sources of stress for the pigs [59], which may then weaken immune response and increase disease susceptibility. Regrouping also enhances the probability of pathogen transmission and frequent movements in subsequent facilities increases the opportunities for exposure to residual infectious agents. Ultimately, the intermingled non-infectious factors related to management practices and herd type strongly influence disease transmission pattern and severity and, in turn, herd performance.

## Conclusions

Risky herd profiles were hereby identified in regard to the technical performance of swine herds. Herd management and viral infections significantly influenced the performance levels of the swine herds included in this study. Areas for improvement related to management practices are available for farmers and those involved in herd health management and performance. Improvement of management practices and reduction in the occurrence of viral infections should significantly contribute to higher herd performance levels and thus farm profitability.

## Additional file

Additional file 1 Table S1 Definition and distribution of the explanatory variables used to assess the factors associated with the levels of growing-finshing performance (41 herds, France, *p*-value from the univariate analysis) (DOCX 28 kb)

## Abbreviations

95% CI: 95% Confidence interval
ADG: Average daily weight gain
CSW: Carcass slaughter weight
FCR: Feed conversion ratio
MORT: % of mortality from 8 to 115 kg
OR: Odds-ratio
PCV2: Porcine circovirus type 2
PRRSV: Porcine Reproductive and Respiratory Syndrome Virus
