# Supplementary material for: Factors associated with the growing-finishing performances of swine herds: an exploratory study on serological and herd level indicators
Source: Porcine Health Manag. 2018 Mar 22;4:6. doi: 10.1186/s40813-018-0082-9 (PMC5863451; doi:10.1186/s40813-018-0082-9)
Supplement: Supplementary file 1 — Table S1 Definition and distribution of the explanatory variables used to assess the factors associated with the levels of growing-finshing performance (41 herds, France, p-value from the univariate analysis) (DOCX 28 kb) [file 40813_2018_82_MOESM1_ESM.docx]

**Table S1: Definition and distribution of the explanatory variables used to assess the factors associated with the levels of growing-finshing performance** (41 herds, France, p-value from the univariate analysis)

| **Definition of the variables** | **% herds per level** | **% herds with low performances per level** | **p-value** |
| --- | --- | --- | --- |
| ***General characteristics of the herd and management*** | | | |
| **Herd type** |  |  | 0.00 |
| Farrow-to-finish | 48.78 | 65 |  |
| Growing-finishing | 51.22 | 19.05 |  |
| **Interval between successive batches (weeks)** |  |  | 0.04 |
| ≤ 3 | 42.5 | 58.82 |  |
| ≥ 4 | 57.5 | 26.09 |  |
| **Batch mingling in the nursery and/or finishing stages** | | | 0.11 |
| No | 82.93 | 35.29 |  |
| Yes | 17.07 | 71.43 |  |
| **Pen constitution in the nursery stage** |  |  | 0.24 |
| according to liveweight of the piglets | 80.49 | 36.36 |  |
| according to the litter ranking (dams' age) | 19.51 | 62.5 |  |
| **Building consistency between the nursery and finishing stage** | | | 0.00 |
| No | 36.59 | 73.33 |  |
| Yes | 63.41 | 23.08 |  |
| **Homogeneity of the pigs during the growing finishing steps** | |  | 0.07 |
| Yes | 12 | 0 |  |
| +/- | 73 | 40 |  |
| No | 15 | 83 |  |
| **Regular clinical signs of illness in growers and finishers** | |  | 0.00 |
| No | 48.78 | 20 |  |
| Yes | 51.22 | 61.9 |  |
| **Feed medication during the second half of the nursery stage** | |  | 0.71 |
| No | 75.61 | 38.71 |  |
| Yes | 24.39 | 50 |  |
| ***Vaccination Schedule*** |  |  |  |
| **Previous piglets vaccination against PCV2** |  |  | 0.59 |
| No | 48.78 | 35 |  |
| Yes | 51.22 | 47.62 |  |
| **Vaccination of the sows against PCV2** |  |  | 0.68 |
| No | 68.29 | 39.29 |  |
| Yes | 31.71 | 46.15 |  |
| **Vaccination of the gilts against PCV2** |  |  | 0.62 |
| No | 42.5 | 47.06 |  |
| Yes | 57.5 | 39.13 |  |
| **Vaccination of the gilts and/or sows against PCV2** | |  | 0.73 |
| No | 43.9 | 44.44 |  |
| Yes | 56.1 | 39.13 |  |
| **At least one vaccination of the growers or finishers** | |  | *0.02* |
| No | 24.39 | 20 |  |
| Yes | 75.61 | 48.4 |  |
| **Vaccination of the piglets against *Mycoplasma hyopneumoniae*** | |  | *0.09* |
| No | 26.83 | 18.18 |  |
| Yes | 73.17 | 50 |  |
| **Age at *Mycoplasma hyopneumoniae* vaccination** |  |  | 1.00 |
| ≤ 4 weeks old | 80 | 50 |  |
| ≥ 5 weeks old | 20 | 50 |  |
| **Vaccination of the piglets against Porcine Reproductive and Respiratory Syndrome (PRRS)** | | | *0.05* |
| No | 80.49 | 33.33 |  |
| Yes | 19.51 | 75 |  |
| ***Serological status against viral and bacterial pathogens*** | |  |  |
| **% of pigs seropositive to** *Lawsonia intracellularis* **after 16 weeks of age** |  |  | 0.48 |
| < 60% | **26.83** | **54.55** |  |
| ≥ 60% | **73.17** | **36.67** |  |
| **Pigs seropositive to *Lawsonia intracellularis*** |  |  | 0.85 |
| before 16 weeks of age | **51.22** | **42.86** |  |
| after 16 weeks of age | **48.78** | **40** |  |
| **% of finishing pigs seropositive to** *Lawsonia intracellularis* | | | 0.68 |
| < 50% | **60.98** | **44** |  |
| ≥ 50% | **39.02** | **37.5** |  |
| **Serological status to** *Mycoplasma hyopneumoniae* |  |  | 0.60 |
| Negative | **55** | **36.36** |  |
| Positive (at least one positive sample) | **45** | **44.44** |  |
| **Serological profile to** *Mycoplasma hyopneumoniae* | |  | 0.80 |
| Negative | **55** | **36.36** |  |
| Positive before 16 weeks of age (at least one positive sample) | **37.5** | **46.67** |  |
| Positive after 16 weeks of age (at least one positive sample) | **7.5** | **33.33** |  |
| **% of pigs with antibodies against** *Mycoplasma hyopneumoniae* before 16 weeks of age | | | 1.00 |
| ≤20% | **80.49** | **42.42** |  |
| >20% | **19.51** | **37.5** |  |
| **% of pigs with antibodies against** *Mycoplasma hyopneumoniae* before 16 weeks of age | | | 1.00 |
| ≤10% | **73.17** | **40** |  |
| >10% | **26.83** | **45.45** |  |
| **% of pigs with antibodies against** *Mycoplasma hyopneumoniae* after 16 weeks of age | | | 0.70 |
| ≤10% | **80.49** | **39.39** |  |
| >10% | **19.51** | **50** |  |
| **% of pigs with antibodies against** *Mycoplasma hyopneumoniae* **during the finishing phase** | | | 1.00 |
| ≤10% | **78.05** | **40.63** |  |
| >10% | **21.95** | **44.44** |  |
| **% of pigs with antibodies against swine Influenza A virus** | | | 0.33 |
| ≤20% | **56.1** | **34.78** |  |
| >20% | **43.9** | **50** |  |
| **% of pigs with antibodies against swine Influenza A virus** | | | 0.18 |
| ≤80% | **70.73** | **34.48** |  |
| >80% | **29.27** | **58.33** |  |
| **Serological status to swine Influenza A virus** |  |  | 0.58 |
| Negative | **46.34** | **36.84** |  |
| Positive (at least one positive sample) | **53.66** | **45.45** |  |
| **Antibodies against PRRSV before 16 weeks of age** | | | 0.05 |
| No | **80.49** | **33.33** |  |
| Yes (at least one positive pool) | **19.51** | **75** |  |
| **Antibodies against PRRSV after 16 weeks of age** |  |  | 0.02 |
| No | **56.1** | **26.09** |  |
| Yes (at least one positive pool) | **43.9** | **61.11** |  |
| **Serological status to PRRSV** |  |  | 0.03 |
| Negative | **56.1** | **26.09** |  |
| Positive (at least one positive pool) | **43.9** | **61.11** |  |
| **Serological profile to PRRSV** |  |  | 0.04 |
| Negative | **56.1** | **26.09** |  |
| Positive before 16 weeks of age (at least one positive pool) | **19.51** | **75** |  |
| Positive after 16 weeks of age (at least one positive pool) | **24.39** | **50** |  |
| **% of pigs with antibodies against Porcine Circovirus Type 2 (PCV2) before 16 weeks of age** | | | 0.51 |
| ≤50% | **29.27** | **50** |  |
| >50% | **70.73** | **37.93** |  |
| **% of pigs with antibodies against Porcine PCV2 after 16 weeks of age** | | | 0.11 |
| ≤70% | **19.51** | **12.5** |  |
| >70% | **80.49** | **48.48** |  |
| **Anti-PCV2 IgG antibody titers >5000 ELISA units before 16 weeks of age** | | | 0.21 |
| No | **85.37** | **34.17** |  |
| Yes (at least one pig) | **14.63** | **66.67** |  |
| **Anti-PCV2 IgG antibody titers >5000 ELISA units after 16 weeks of age** | | | 0.10 |
| No | **31.71** | **23.08** |  |
| Yes (at least one pig) | **68.29** | **50** |  |
| **Anti-PCV2 IgG antibody titers >5000 ELISA units during the fattening phase** | | | 0.17 |
| No | **31.71** | **23.08** |  |
| Yes (at least one pig) | **68.29** | **50** |  |
| **>10% of pigs with a SERELISA® titer >5000 ELISA Units for antibodies against PCV2** | | | 0.05 |
| No | **41.46** | **23.53** |  |
| Yes | **58.54** | **54.17** |  |
| **>20% of pigs with a SERELISA® titer >5000 ELISA Units for antibodies against PCV2** | | | 0.28 |
| No | **51.22** | **33.33** |  |
| Yes | **48.78** | **50** |  |
